# 

**Published:** 1888-08-25

**Authors:** 


CONTENTS.

**A Medical Plan of Campaign** - 333

**How to Grow Beautiful** - 334

**Hospital Finance and Economy.**—By a HOSPITAL SUPERINTENDENT - 335

**Round About the Asylums.**—XIII.—By a MEDICAL SUPERINTENDENT - 336

**False Issues and Fanciful Facts.**—A Review - 337

**The Doctor's Pulpit.**—Voluntary Imbecility - 339

**Notes and News** - 340

**Everybody's Page** - 342

**Annotations.**—Ambulances Wanted.—The Weather Notwithstanding.—The Value of a Little Medical Knowledge - 343

**Climate and Suicide** - 344

**An Ishmaelite.** By LESLIE KEITH, Author of "The Chilcotes," "East and West," etc. - 345

**Swiss Small-pox Statistics** - 346

**The Student's Column** - 347

**Good-natured Committees** - 347

**Amusements with Profit for all Ages.**—For Men and Wo nen.—Children's Corner.—Puzzles, etc. - 348

EXTRA SUPPLEMENT.—THE NURSING MIRROR.

En Passant —A Natural Sequence !—Poor Student !—The Duty of Thankfulness.—The Question of Uniform.—Two Notes from America.—Watching Lunatics.—A Catechism for Nurses.—Courage - lxxxi
A Touching Story of Gratitude - lxxxiii
Original Articles.—A Nurse's Progress.—From Application to Certification.—Paper III. Certification - lxxxii
Everybody's Opinion.—Censoriousness.—Small Hospitals for Inexperienced Probationers.—The Cause of Weak Ankles - lxxxiii
Examination Questions.—Vacancies.—Notes and Queries - lxxxiv

